# Cellular gatekeepers of infected wounds: the dual role of adipocytes in coordinating immunity and regeneration

**DOI:** 10.3389/fimmu.2026.1793958

**Published:** 2026-03-27

**Authors:** Hang Yan, Ying Wang, Xigetu He, Yunxuan Li, Linjing Yang, Jintao Yang, Xingke Meng, Congxiao Peng, Xiaofan Zhang, Aobuliaximu Yakupu, Yin Wen, Qi He, Mulan Qahar

**Affiliations:** 1 The Collaborative Innovation Center of Tissue Damage Repair and Regeneration Medicine, Zunyi Medical University, Zunyi, Guizhou, China; 2 Department of Neurology, First People’s Hospital, Zunyi, Guizhou, China; 3 Department of Dermatology, International Mongolian Hospital of Inner Mongolia, Hohhot, China; 4 Taizhou Hospital of Zhejiang Province, Wenzhou Medical University, Taizhou, Zhejiang, China; 5 Department of Burns & Wound Care Center, the Second Affiliated Hospital of Zhejiang University, School of Medicine, Hangzhou, China

**Keywords:** adipocytes, AMPs, fat grafting, immune regulation, infected wounds, tissue regeneration, wound healing

## Abstract

Chronically infected wounds continue to pose a major challenge in clinical practice. Their persistence reflects the convergence of sustained microbial colonization, inappropriate or prolonged immune activation, and insufficient tissue regenerative capacity, which together establish a self-reinforcing pathological environment. In routine clinical management, approaches centered on debridement and antimicrobial therapy often fail to achieve durable healing, indicating that key regulatory mechanisms governing wound repair remain insufficiently understood. Accumulating evidence has drawn attention to adipocytes as an integral, yet previously underrecognized, component of the infected wound microenvironment. Beyond their established metabolic role, adipocytes respond rapidly to infectious injury and contribute to early host defense through several coordinated processes. These include migration-associated morphological remodeling that provides transient coverage at the wound margin, local production of antimicrobial peptides that directly restrict microbial growth, and secretion of complement components and chemotactic mediators that shape the recruitment and function of innate immune cells such as neutrophils and macrophages. As infection is brought under control, adipocyte activity shifts toward supporting tissue repair, with paracrine signals influencing fibroblast behavior, angiogenesis, re-epithelialization, and granulation tissue development. On this basis, this review summarizes current knowledge of adipocyte functions across distinct phases of infected wound healing and discusses emerging translational strategies that leverage adipocyte biology, including refinements in autologous fat grafting and the development of adipose-derived biomaterials. A clearer understanding of adipocyte functional plasticity may help guide therapeutic approaches that integrate immune regulation with regenerative processes, thereby improving outcomes in chronic, non-healing wounds.

## 1 Introduction

Infected wounds represent a common yet formidable challenge in clinical practice and are often difficult to treat effectively. Their pathophysiology is driven by a vicious interplay between persistent pathogen invasion and disruption of the host immune microenvironment, reflecting the combined consequences of impaired pathogen clearance and dysregulated tissue regeneration (1, 2). Clinically, infectious wounds are frequently encountered in patients with diabetic foot ulcers, post-burn infections, venous ulcers, and secondary infections following trauma. These wounds are typically characterized by delayed healing and recurrent infection and may, in severe cases, progress to systemic complications such as sepsis, resulting in concurrent local tissue damage and systemic functional impairment (2, 3). Epidemiological data indicate that chronic wounds affect approximately 2% of the population in the United States, with prevalence rates reaching up to 6% in certain regions, such as Wales. The associated annual healthcare expenditures are estimated to be in the tens of billions of US dollars, imposing a substantial burden on healthcare systems and markedly diminishing patients’ quality of life (3, 4). Taken together, these challenges underscore the need to clarify the cellular and molecular mechanisms governing infected wound healing and to develop innovative therapeutic strategies.

To frame the subsequent discussion, in this review, infected wounds denote wounds in which microbial burden elicits a host inflammatory response that delays healing, consistent with the contemporary wound-infection continuum. Clinically, this spectrum can be stratified into: (i) biofilm-dominant chronic ulcers (persistent inflammation and antimicrobial tolerance), (ii) acute bacterial wound infection (rapid planktonic expansion with overt local/systemic signs), and (iii) critical colonization (increased bioburden that delays healing without classical infection signs; often discussed as covert/local infection in consensus frameworks) (5, 6). Recent cross-species studies have prompted a re-examination of the role of adipocytes in infection-associated wound repair. In mammals, adipose tissue is generally categorized into white and brown depots, reflecting their predominant roles in energy storage and thermogenic energy dissipation, respectively (7, 8). Traditionally, adipose tissue has been viewed as a multifunctional organ that provides mechanical cushioning and structural support (9), serves endocrine roles through the secretion of leptin, adiponectin, and other metabolic hormones, and acts as a dynamic reservoir for energy storage and mobilization (10, 11). In addition, subcutaneous adipose tissue contributes to thermal insulation and adaptation to cold environments (12). For instance, subcutaneous adipocytes can mount rapid responses to cutaneous infections by secreting AMPs that directly enhance host defense (13); *in vivo* imaging in Drosophila further demonstrates that fat-body cells can migrate directionally to wound margins and extend broad pseudopodia, thereby effecting a transient physical seal of the wound bed (14). These observations underscore the diverse functional roles of adipocytes during infected wound repair. In this review, we examine how adipocyte behavior evolves over time during infection-associated wound repair. Particular attention is given to the dynamic changes in adipocyte function across distinct phases of the healing response. We also examine translational strategies centered on adipocytes, including cell-based interventions and bioengineering approaches. In contrast to earlier work that largely emphasized the anatomical features of dermal white adipose tissue or pathways related to fibrosis, the present review integrates mechanistic findings with emerging therapeutic applications and seeks to inform future strategies for the management of chronic infected wounds.

**Figure 1:**
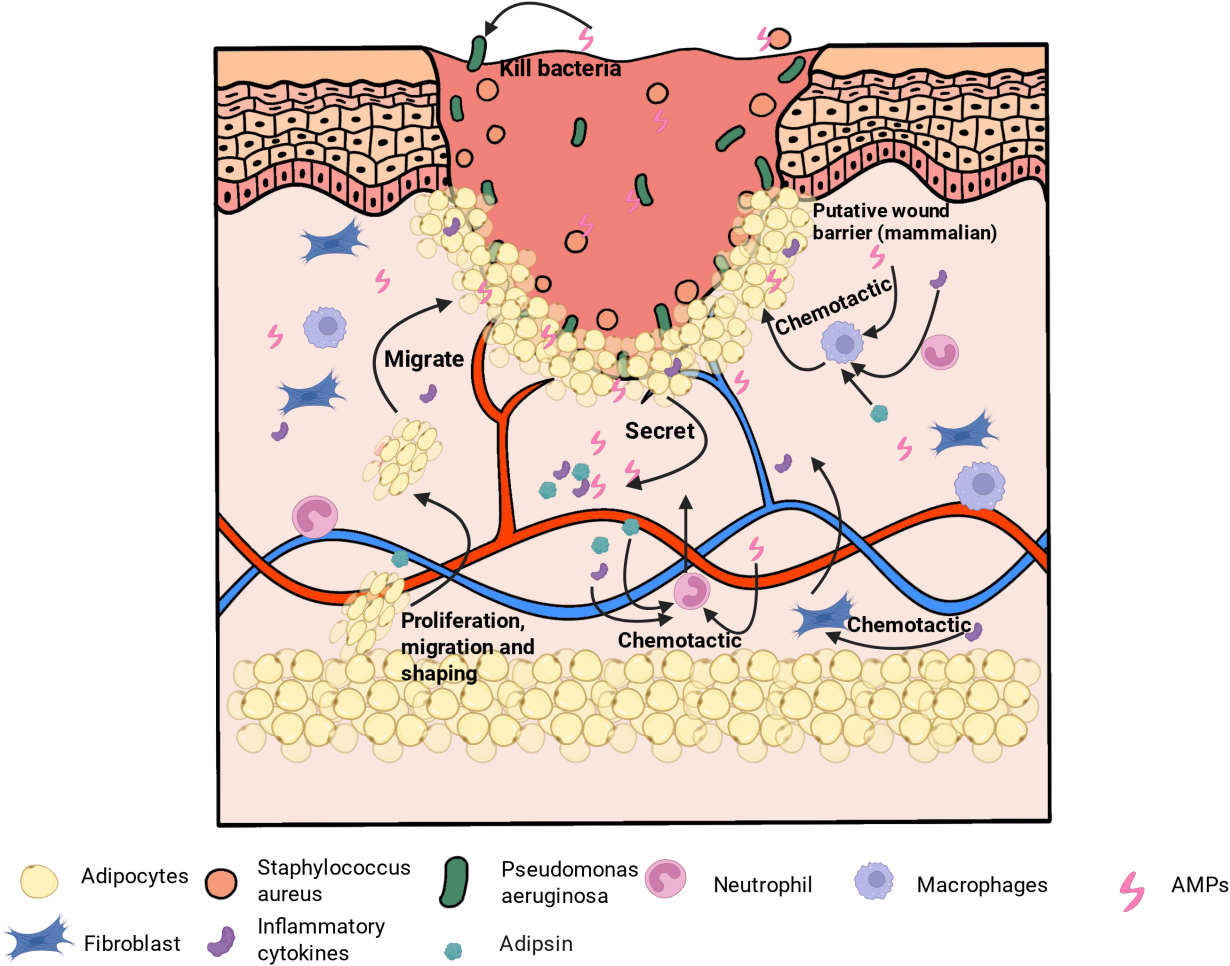
Roles of adipocytes during infected wound healing. Following pathogen invasion, adipocytes undergo morphological remodeling and migrate toward the wound margin, contributing to transient physical coverage and the secretion of immune mediators, including antimicrobial peptides, interleukin-8, and complement factors. Concurrently, enhanced lipolysis and interactions with fibroblasts and other stromal cells support granulation tissue formation and tissue repair.

The search employed the following key terms: (“adipose tissue” OR adipocyte) AND (“wound healing” OR “wound repair”) AND (infection OR “infected wound” OR “bacterial infection”) AND (immun* OR inflammation OR antimicrobial). Included studies focused on adipocytes or adipose tissue in infected or bacterial skin wound models, specifically addressing immune regulation, antimicrobial defense, metabolism, or regeneration. Only original research articles, reviews, and meta-analyses published in English were considered. Exclusions comprised studies on adipose tissue in purely metabolic contexts unrelated to wound healing, those involving non-cutaneous tissues, wound models lacking an infectious or immune component, and non−English publications, conference abstracts, commentaries, and editorials. Search results were screened by title and abstract, followed by full−text review of potentially relevant articles. The overall roles of adipocytes during infected wound healing are summarized in **Figure 1**.

## 2 Proactive defense by adipocytes in infected wounds: barrier formation and immune coordination

Major adipocyte-derived mediators involved in infected wound repair are summarized in **Table 1**. The early physical barrier in mammalian wound repair has traditionally been attributed to platelet–fibrin clot formation, followed by re-epithelialization to restore epidermal integrity (15). However, observations from infected wound models indicate that this framework does not fully capture the range of cellular responses involved. In a murine Staphylococcus aureus skin infection model, the subcutaneous adipose layer expands rapidly at early time points and becomes a prominent source of antimicrobial peptides, which directly enhance local host defense (13). On this basis, adipocytes are increasingly recognized as active participants in the pathophysiology of infected wounds rather than passive bystanders. Experimental studies indicate that their contributions unfold in a coordinated, stage-dependent manner. During the early phase, mature adipocytes migrate toward the wound margin and undergo pronounced morphological remodeling, collectively forming a transient physical barrier. As the response progresses, adipocytes release effector molecules, including antimicrobial peptides and adipsin, which influence the recruitment and activation of neutrophils, macrophages, and other innate immune cells, thereby facilitating pathogen clearance. At later stages, adipocytes engage additional immune-regulatory mechanisms, such as antigen presentation, linking innate immune activation to adaptive immune responses and supporting the transition from inflammation to tissue regeneration.

**Table 1:** Major adipocyte-derived mediators involved in infected wound repair.

Factor	Mechanism of action	Key references
Antimicrobial peptides (AMPs)	Direct bactericidal activity; promote re-epithelialization; augment local innate immune defense	Zhang et al., 2015; Franz et al., 2018 (13, 14)
adipsin	Activates the complement cascade (alternative pathway); enhances opsonophagocytic clearance by neutrophils and macrophages	King et al., 2024 (47)
IL-8 (CXCL8)	Chemotactic recruitment of neutrophils and other leukocytes to the wound; promotes leukocyte activation and migration	Cambier et al., 2023 (56)
Adiponectin	Regulates macrophage polarization (drives M1→M2 shift); facilitates resolution of inflammation and immune-mediated tissue repair	Sohn et al., 2018 (60)
Leptin	Accelerates keratinocyte migration and proliferation; enhances the re-epithelialization process	Tadokoro et al., 2015 (92)

### 2.1 Migration to the wound edge and formation of a physical barrier

Early work generally regarded adipocytes as largely immobile cells, in contrast to the dynamic migratory behavior typical of immune cells *in vivo* (16). This assumption was later revised by the study of Franz and colleagues provided the first *in vivo* imaging evidence that fat-body cells in Drosophila larvae and pupae relocate to sites of tissue injury in response to injury signals, forming a transient barrier. Using a laser-induced epidermal wound model, they showed that fat-body cells, which are functionally comparable to mammalian adipocytes, dispersed within the hemocoel in response to injury signals and progressively accumulated at the wound site within several hours (14). Evidence from mammalian systems has since supported a similar phenomenon. Studies from the Horsley group identified a subset of adipocytes at the margins of murine full-thickness wounds that display morphological changes consistent with active migration, although direct evidence for long-range migration in mammals is still lacking. These cells adopt a flattened morphology and extend slender cellular protrusions, allowing them to traverse interstitial spaces within the wounded tissue (17). Although real-time imaging of long-range, single-adipocyte migration in mammals is still lacking, these morphological changes indicate that adipocytes can be activated by injury signals and undergo directed relocalization. In summary, Drosophila models provide direct *in vivo* imaging evidence of fat-body cell migration and barrier formation, whereas mammalian studies rely on morphological and positional assessments, with direct imaging evidence still absent.

With respect to migratory mechanisms, mature mammalian adipocytes can adopt a motile phenotype and infiltrate the wound following injury, which is associated with the upregulation of cytoskeletal remodeling programs (17). Available data indicate that PDGF and TGF-β signaling in infected wounds enhances adipocyte plasticity, endowing them with greater migratory and relocalization capacities (18). In contrast, *Drosophila* fat-body cells advance through the hemolymph via an adhesion-independent, actin-driven ‘swimming’ mode (14), reminiscent of the adhesion-free migration utilized by immune cells in confined 3D matrices (19). At the molecular level, rear cortical contraction mediated by the Rho1–ROCK–myosin II pathway, along with Dia-dependent actin polymerization and Cdc42/Rac1–Arp2/3/WASP-coordinated regulation, collectively drives the directional movement of fat-body cells (20). In mammals, current observations include lipid-droplet remodeling, cytoskeletal reorganization, and shape deformation in adipocytes within infected wounds—changes that reduce effective cell volume and increase deformability to facilitate movement through dense extracellular matrices (17, 21). Therefore, the regulatory network governing adipocyte motility in mammals remains to be fully delineated.

Adipocytes that infiltrate infected wounds can extend broad, lamellipodia-like sheets to bridge intertissue gaps, creating an early, transient seal that is critical for limiting microbial ingress and reducing fluid loss (22). In small wounds, keratinocyte migration and purse-string contraction may suffice for closure; however, in larger or infected wounds, epidermal cells alone are often inadequate for immediate sealing (15). In *Drosophila*, fat-body cell spreading provides a form of “backup sealing” for the lesion (14). In mammals, supporting evidence primarily arises from the plastic changes observed in dWAT adipocytes: these cells can dedifferentiate into flattened, highly extendable fibroblast-like cells (23), migrate toward the wound center, spread, secrete extracellular matrix, and interconnect to form a barrier-like scaffold that provides structural support and a substrate for re-epithelialization from above (24).

Three caveats merit emphasis. First, direct imaging evidence of mammalian adipocytes forming extensive lamellipodial sheets over the wound gap remains absent. Second, although some studies suggest that adipocytes may dedifferentiate into fibroblast-like cells, lineage tracing and single-cell RNA sequencing indicate that these cells do not fully transdifferentiate into scar-forming fibroblasts, maintaining lineage restriction (17). Third, in murine models, lineage tracing is typically achieved using Cre–loxP–based genetic reporter systems that enable permanent cell labeling and fate mapping during wound repair (25). In humans, direct genetic lineage tracing is not feasible (26); instead, lineage relationships are inferred using single-cell RNA sequencing combined with trajectory analysis or RNA velocity modeling, which provide high-resolution but indirect evidence of cellular identity and plasticity (27). Despite these controversies, the role of adipocytes as early physical scaffolds and cellular sources within the wound bed is increasingly acknowledged, challenging the traditional notion that mature adipocytes are terminally differentiated and functionally inert.

**Figure 2:**
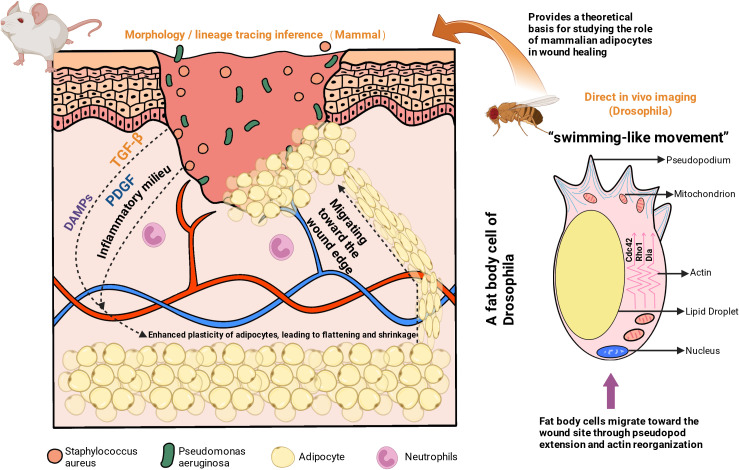
Migration of adipocytes and formation of physical barrier in infected wounds. In Drosophila, adipose cells respond to injury by reorganizing their cytoskeleton and migrating toward the wound edge via an actin-driven ‘swimming’ mode, forming a transient physical barrier, as directly observed in real-time *in vivo* imaging. In mammalian infected wounds, adipocytes display similar migratory features, such as cell flattening, protrusion formation, and lipid-droplet remodeling, though direct real-time *in vivo* imaging remains limited. These responses are regulated by pathways like PDGF and TGF-β signaling, highlighting the role of adipocyte-directed migration in early wound repair, although direct *in vivo* visualization in mammals is still lacking.

Functionally, this transient physical barrier is crucial for defense and homeostasis. It prevents deeper penetration of bacteria and foreign material before immune cells gain full control of the infection (28, 29). At the same time, this coverage limits evaporative fluid loss and preserves a moist microenvironment—similar to a “biological bandage”—that promotes cell migration and tissue regeneration (30). A cross-species overview of adipocyte migration and physical barrier formation is shown in **Figure 2**.

### 2.2 Secretion of AMPs and immune mediators, and intercellular coordination

#### 2.2.1 Antimicrobial peptide production and function

In addition to forming a physical barrier, adipocytes (including newly differentiated fat cells) serve as crucial innate effector cells during early infection by secreting AMPs that enhance local defense mechanisms. Traditionally, it was thought that wound anti-infective activity relies mainly on immune cells (e.g., neutrophils that release antimicrobial molecules, and macrophages that perform phagocytosis) as well as on keratinocyte-derived AMPs (31–33). However, this paradigm has been challenged by emerging evidence. In a murine model of Staphylococcus aureus skin infection, Zhang et al. demonstrated that the early thickening of the subcutaneous adipose layer is indicative of the proliferation and differentiation of adipocyte precursors (adipogenesis), rather than merely the hypertrophy of mature adipocytes. Both newly formed and mature adipocytes significantly upregulate and secrete cathelicidin-family AMPs (CRAMP in mice; LL-37 in humans), thereby enhancing local antimicrobial defense (13). Recent studies indicate that PPAR−γ acts as a key transcription factor driving adipogenesis: in the context of skin infection, dermal fibroblasts may be induced to express PPAR−γ and differentiate into preadipocytes/adipocytes, which in turn robustly up−regulate Cathelicidin expression, contributing to local antimicrobial defense (34). This “adipogenesis–AMP” axis likely represents a critical early barrier to infection.

Cross-species studies further elucidate this role. In rainbow trout, infection or exposure to pathogen-associated molecular patterns (PAMPs) leads to the upregulation of multiple antimicrobial AMPs, such as cathelicidin-2 and hepcidin, within visceral adipose tissue (35); similarly, in *Drosophila*, fat-body cells that migrate to the wound site induce AMPs, including Attacin and Drosomycin, which help to restrain bacterial load (21). Collectively, across species from insects to mammals, adipocytes and their homologous tissues are rapidly activated in response to infectious challenges and serve as significant sources of AMPs that complement the traditional defenses provided by immune and epithelial cells.

However, this beneficial response carries a “double-edged sword” risk in chronic wounds. Prolonged or dysregulated AMP activity (e.g., sustained LL-37) can itself drive chronic inflammation, disrupt tissue regeneration, and promote fibrosis, thereby impairing wound closure. This highlights the critical importance of the temporal regulation of adipocyte AMP responses: they must be robustly activated for early defense but appropriately downregulated to permit the transition to subsequent healing phases (36).

Mechanistically, AMP induction in adipocytes occurs as a direct response to pathogen-associated molecular patterns (PAMPs). Bacterial or fungal components (such as lipoteichoic acid, peptidoglycan, lipopolysaccharide) activate TLR2/TLR4 on the surface of adipocytes, initiating the PI3K/STAT3 signaling cascade, which amplifies the cellular response in coordination with the antagonism of the TGF-β pathway, upregulating the transcription of the CAMP gene to produce more antimicrobial peptides (37, 38). Additionally, local 1, 25-dihydroxyvitamin D_3_ (1, 25(OH)_2_D_3_) enhances CAMP transcription through the binding of the vitamin D receptor (VDR) to vitamin D response elements within the CAMP promoter (39); fungal signals can also activate the fibroblast growth factor receptor–mitogen-activated protein kinase (FGFR–MEK–ERK) pathway, linking external infection signals to adipogenic programs (34). Newly synthesized cathelicidin is secreted as the precursor hCAP18, which is subsequently processed in the wound microenvironment by proteases such as neutrophil proteinase 3 or keratinocyte-derived serine proteases to generate bioactive LL-37 peptides (40). Activated LL-37 can be selectively delivered to sites of infection via extracellular vesicles, such as exosomes (41).

**Figure 3:**
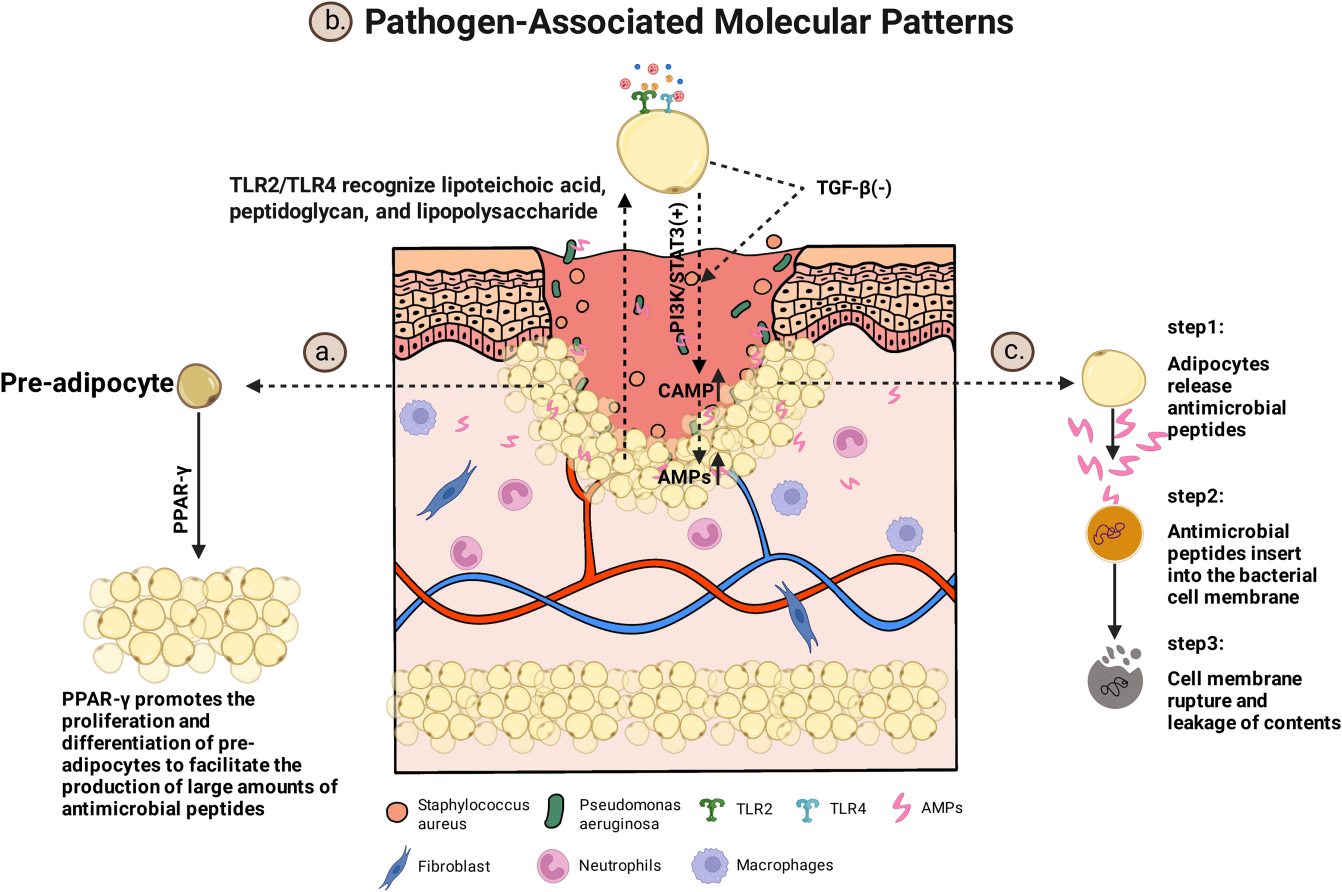
Role of adipocyte-derived antimicrobial peptides in infected wounds. Pathogen-associated molecular patterns activate TLR2 and TLR4 on adipocytes, promoting PPAR-γ–dependent adipogenesis and antimicrobial peptide production while attenuating TGF-β–mediated inhibitory signals. Adipocyte-derived antimicrobial peptides are released into the wound microenvironment, where they exert broad-spectrum antimicrobial activity against invading pathogens.

Unlike transiently recruited leukocytes, adipocytes constitute a stable, residential component of local innate immunity. Owing to their large size, with diameters reaching approximately 100 μm, and their long lifespan, adipocytes are capable of sustaining AMP production from the early stages of infection through subsequent tissue repair, thereby providing prolonged local protection (13, 42). Beyond their antimicrobial activity, AMPs such as LL-37 also participate directly in tissue repair by enhancing keratinocyte motility and promoting re-epithelialization, thereby supporting wound closure while limiting microbial expansion (12, 43, 44). Evidence further indicates that the inducibility of this adipogenesis-associated AMP response is shaped by host context. In conditions such as aging or obesity, this pathway appears less responsive, potentially weakening local innate defense in susceptible individuals (45). From a translational standpoint, interventions that reinforce adipocyte immune competence—for example, by improving the local metabolic milieu to sustain AMP production—may represent a feasible strategy to improve healing outcomes in infected wounds. The role of adipocyte-derived antimicrobial peptides in infected wounds is summarized in **Figure 3**.

#### 2.2.2 Adipsin mediated modulation of inflammatory responses in wounds

During wound repair, adipocytes shape the local immune microenvironment in part through the secretion of adipsin. As the rate-limiting protease of the alternative complement pathway, adipsin is abundantly produced by adipocytes at early stages of infection and is associated with local activation of complement (46). This activation supports C3 convertase formation and promotes cleavage of C3 into the effector fragments C3a and C3b, thereby facilitating downstream complement-dependent immune responses within the wound bed (47).

Adipsin contributes to immune regulation through several complement-dependent mechanisms. C3a, via engagement of its receptor, has been shown to support the recruitment and activation of neutrophils and macrophages at wound sites (48), whereas C3b facilitates microbial recognition and clearance by phagocytes through opsonization of pathogen surfaces (49). Together, these mechanisms help establish a local immune environment that is compatible with effective pathogen control while allowing subsequent tissue repair to proceed. In this context, adipocytes function not only as a major source of factor D but also express the C3a receptor (C3aR1), indicating the existence of an autocrine complement-related regulatory loop, often referred to as the “adipocyte–complement–adipocyte” pathway (46, 50, 51). However, if complement activation is excessive or prolonged, sustained C3a/C3b signaling may amplify neutrophil-dominant inflammation and collateral tissue injury, thereby hindering the transition from inflammation to repair in chronic wounds.

Across the course of wound healing, adipocytes contribute to inflammatory regulation in part through the secretion of factor D. Available evidence suggests that, during later stages of repair, this pathway may also participate in the attenuation of inflammatory activity, facilitating a gradual shift in the wound microenvironment from a pro-inflammatory state toward conditions more supportive of tissue restoration (52, 53). Such temporal control of inflammatory intensity is likely important for limiting collateral tissue damage and for enabling the expansion and function of reparative cell populations. From this perspective, targeting adipocyte-derived factor D or components of its downstream signaling network could be considered as a potential approach to support wound repair. Further investigation will be required to clarify how this pathway is modified under pathological conditions and to delineate the mechanisms through which it influences inflammatory resolution and tissue regeneration.

#### 2.2.3 Coordination between adipocytes and neutrophils in antibacterial defense

Neutrophils are among the earliest immune effector cells recruited to wound sites and play an important role in pathogen clearance and the removal of tissue debris (31). Recent studies indicate that adipocytes engage in close functional cooperation with neutrophils through multiple pathways, collectively supporting effective antibacterial responses.

In the direct pathway, adipocyte-derived cathelicidin antimicrobial peptides, such as LL-37, exhibit broad antimicrobial activity and have been shown to promote neutrophil recruitment through FPR2-dependent chemotactic signaling. Moreover, these peptides can enhance neutrophil phagocytic capacity and support the formation of neutrophil extracellular traps (54). Beyond these direct effects, adipocyte-secreted adipsin activates the C3a and C3b pathway, which further supports neutrophil recruitment and complement-mediated opsonophagocytosis (51). These mechanisms link adipocyte-derived antimicrobial and complement responses with neutrophil-mediated host defense and contribute to effective pathogen clearance in infected wounds. Adipocytes may also contribute to early neutrophil recruitment through the secretion of interleukin-8 (IL-8, CXCL8). Previous studies have shown that human adipose tissue and adipocytes are capable of producing IL-8 in response to inflammatory stimuli (23, 55), and IL-8 is well recognized as a potent chemoattractant for neutrophils (56). Given that adipocytes are activated shortly after cutaneous injury (44), they may represent an important local source of IL-8 within the wound microenvironment, thereby supporting early neutrophil infiltration and activation.

These coordinated interactions underscore the involvement of adipocytes in innate immune responses and help clarify how inflammatory activity is regulated during wound healing. By engaging neutrophils through both direct and indirect pathways, adipocytes participate in integrated host defense processes that contribute to effective protection against infection.

While this coordinated response is essential for early pathogen control, it embodies a “double-edged sword.” If unresolved, persistent neutrophilic activity—characterized by excessive NET formation, protease release, and ROS production—can cause collateral tissue damage and delay healing. Furthermore, the sustained activation of the very adipocyte-derived pathways that initiate this defense (e.g., AMP and complement signaling) can feed into a cycle of chronic inflammation, tissue destruction, and fibrosis, ultimately impeding the transition to the tissue regeneration phase (57, 58).

#### 2.2.4 Coordination between adipocytes and macrophages in debridement and inflammatory resolution

During wound repair, mature adipocytes and macrophages interact bidirectionally, shaping both inflammatory activity and subsequent tissue regeneration (25, 59). The nature of this crosstalk varies over time and is characterized by stage-specific functional roles.

Early after infection, adipocytes contribute to macrophage recruitment and modulation via several routes. Free fatty acids generated by lipolysis provide metabolic substrates and also function as signals that promote macrophage trafficking into the wound site (25, 60). Adipocyte-derived adiponectin further shapes macrophage responses by biasing these cells toward an M2-like, anti-inflammatory state, while supporting phagocytic function and limiting excessive inflammatory signaling (61). In line with these effects, studies using adipocyte-conditioned media have reported increased macrophage motility and activation, indicating that adipocytes directly participate in guiding macrophage recruitment within the wound microenvironment (60).

During the removal of necrotic tissue, adipocytes appear to support macrophage activity through both structural and molecular contributions. Changes in adipocyte morphology, together with the extension of pseudopodial-like processes, may help displace and fragment necrotic material, thereby facilitating subsequent macrophage phagocytosis (14). In parallel, adiponectin released by adipocytes has been reported to act as an opsonizing factor that promotes the recognition and clearance of apoptotic cells (62). By supporting efficient debris removal at this stage, these adipocyte-associated processes may help limit the persistence of inflammatory stimuli and create conditions more favorable for later phases of tissue repair.

As healing advances, mature adipocytes continue to influence macrophage behavior through paracrine interactions. Evidence suggests that extracellular vesicles released by adipocytes carry bioactive signals capable of biasing macrophage polarization away from a pro-inflammatory profile and toward a reparative state (63, 64). Such changes in macrophage phenotype are thought to accompany the gradual resolution of inflammation and to coincide with processes such as granulation tissue development and re-epithelialization during wound repair (65).

Interactions between adipocytes and macrophages are mediated by several signaling routes. Beyond adiponectin-dependent effects, mature adipocytes release cytokines, including IL-6 and TGF-β, that influence macrophage activation and functional state (66). Conversely, macrophages activated within the wound environment secrete inflammatory mediators such as TNF-α and IL-1β, which can alter adipocyte metabolism and secretory behavior (67). Together, these reciprocal signals establish a tightly coupled regulatory relationship between the two cell types.

Altered communication between adipocytes and macrophages has been associated with impaired healing responses in several types of chronic wounds. In metabolic disorders such as diabetes, disruption of this regulatory interplay is accompanied by defects in macrophage phenotypic switching, which can delay or derail the normal sequence of tissue repair events (68). These observations suggest that restoring appropriate adipocyte–macrophage crosstalk may offer a strategy to improve healing outcomes in otherwise non-resolving wounds.

Throughout the wound repair process, coordinated interactions between adipocytes and macrophages contribute to the regulation of inflammation, efficient removal of damaged tissue, and the progression toward regeneration. Growing evidence indicates that this intercellular dialogue is an important determinant of repair outcomes. A more detailed understanding of the molecular pathways governing adipocyte–macrophage communication may therefore help identify actionable targets for improving wound healing in pathological settings.

#### 2.2.5 Targeted regulation of T-cells: antigen-presentation pathways

Within the dermal adipose microenvironment, adipocytes possess the capacity to link innate and adaptive immune responses through antigen presentation, a function mediated by two principal pathways. First, in response to inflammatory cues such as interferon gamma, adipocytes upregulate major histocompatibility complex class II molecules and co-stimulatory signals, enabling them to present peptide antigens to CD4^+^ T cells (69). This suggests that, within a highly inflammatory wound environment, adipocytes can act in coordination with conventional antigen presenting cells, such as adipose tissue macrophages and dendritic cells, to collectively shape CD4^+^ T cells and accelerate the targeted activation and expansion of helper T cells.

Second, mature adipocytes constitutively express CD1d, a molecule that presents endogenous or microbe-derived lipid antigens to invariant natural killer T (iNKT) cells (70). However, while this antigen-presenting capacity is well-documented in adipose tissue immunology, direct evidence for its occurrence in infected wound environments remains limited. Therefore, its role in wounds should be considered a plausible extension of adipose immunology, pending verification in relevant wound models. Upon activation, invariant natural killer T cells rapidly produce cytokines that provide positive or negative feedback to modulate local inflammatory responses (71). The physiological relevance of this adipocyte–iNKT cell axis is supported by genetic evidence: mice with adipocyte-specific deletion of CD1d exhibit impaired iNKT cell function and exacerbated adipose tissue inflammation (72).

### 2.3 Summary

Evidence from infected wound models indicates that adipocytes engage in a range of coordinated activities that evolve over the course of repair. During early stages, adipocytes contribute to physical containment and local antimicrobial defense through barrier formation and the release of antimicrobial peptides and complement components. As the response progresses, they interact closely with neutrophils and macrophages to support bacterial clearance and removal of damaged tissue. At later stages, adipocytes participate in shaping adaptive immune responses, in part through antigen presentation, thereby supporting the transition toward tissue reconstruction. Collectively, these observations highlight adipocytes as active participants in wound immunity rather than passive energy-storing cells. Their involvement across innate and adaptive phases suggests that adipocytes influence both the efficiency and trajectory of repair within the infected wound microenvironment. Representative *in vivo*, *in vitro*, and clinical studies investigating adipocyte functions in infected wound repair are summarized in **Table 2**.

**Table 2:** Summary of *in vivo*, *in vitro*, and clinical studies investigating adipocyte functions in infected wound repair.

Evidence type	Model (species) / Cells	Injury / infection context	Adipocyte-related focus	Key immune findings	Key repair/regeneration findings	Reference
In vivo	Mouse	Staphylococcus aureus skin infection	Dermal adipocyte expansion/adipogenesis; cathelicidin (CRAMP/LL-37 axis)	Adipocytes act as an AMP source enhancing antibacterial defense	Links early host defense to improved local control of infection	Zhang LJ et al., Science 2015; doi:10.1126/science.1260972 (13)
In vivo	Drosophila larvae/pupae	Laser-induced epidermal wound ± infection risk	Laser-induced epidermal wound ± infection risk	Motile fat-body cells reduce infection susceptibility; innate defense program	Provides rapid “backup sealing” of wound gap	Franz A et al., Dev Cell 2018 (14)
In vivo	Mouse	Full-thickness skin wound repair	Dermal adipocyte lipolysis; proposed myofibroblast conversion	Immune–metabolic switching during repair; inflammation-resolution coupling	Lipolysis supports efficient repair, granulation progression	Shook BA et al., Cell Stem Cell 2020 (25)
In vitro	Human/mouse adipocytes (primary or differentiated)	PAMP/TLR stimulation (LTA/LPS/PGN)	CAMP/LL-37 induction via TLR2/4	PI3K/STAT3-related induction of AMP expression	Mechanistic bridge for adipocyte antimicrobial function	Hopfinger A et al., Innate Immun 2021 (38)
In vitro	Adipocytes	cfDNA/TNFα stimulation	CAMP gene regulation	Inflammatory cues modulate adipocyte AMP program	Supports “inflammation→AMP” responsiveness	Hopfinger A et al., Int J Mol Sci 2023 (37)
In vivo + in vitro	Mouse + dermal preadipocytes	Candida albicans skin infection	AMP-producing preadipocytes; FGFR–MEK–ERK	Antifungal defense via preadipocyte AMP program	Reinforces infection-specific adipogenic immune response	Hohl TM et al., PLOS Pathog 2023 (34)
Clinical trial / RCT	Human	Acute burn wound management	Autologous fat transfer	(Depending on trial endpoints) reduced inflammation / improved wound milieu	Improved healing outcomes vs controls (trial-specific endpoints)	Abouzaid AM et al., Burns 2022 (101)
Clinical trial	Human	Fat graft survival enhancement	SVF-enriched lipotransfer	Potential immune-compatible autologous enhancement	Improved graft retention (context-specific)	Wufuer M et al., Plast Reconstr Surg 2024 (108)

## 3 Regression of the adipose barrier: a precise switch from immune defense to repair

As infected wounds progress into the proliferative stage, the local environment changes alongside declining inflammation and reduced microbial load. Within this context, regression of the early adipose barrier becomes a characteristic feature of the repair process. Rather than representing simple cell loss, this regression is accompanied by a shift in adipocyte behavior that aligns with changing requirements of the wound. Activities previously oriented toward immune defense give way to functions that support matrix remodeling and tissue closure. Defining the signals that drive this switch and its downstream consequences will be important for understanding how wound repair transitions from pathogen control to structural restoration.

### 3.1 Lipolysis driven regression of the adipose barrier and its role in structural remodeling and energy supply

Regression of the adipose barrier is evident at both histological and molecular levels. In murine injury models, adipocytes located at the wound margin display progressive thinning, cell shrinkage, and a reduction in cell number following the peak of inflammation (25). During this period, these adipocytes enter a highly lipolytic state, characterized by fragmentation of large lipid droplets into smaller structures and the release of fatty acids and other energy substrates that support ongoing repair processes (24). Transcriptomic analyses further show that adipocytes migrating into the wound undergo a coordinated shift in gene expression, moving away from programs associated with lipid synthesis and storage toward pathways enriched for cytoskeletal remodeling and cellular motility. This molecular transition parallels the histological regression of dermal white adipose tissue (dWAT) thickness observed during repair (17). Together, these findings suggest that adipocytes transition from a relatively static energy‐storage role to a more dynamic state that actively supports tissue repair.

The ultimate fate of adipocytes during this process highlights the complexity of their phenotypic flexibility. Some studies have proposed that a subset of adipocytes may undergo dedifferentiation toward fibroblast-like cells that produce extracellular matrix and directly contribute to matrix deposition and wound contraction (25, 73). In contrast, accumulating evidence from lineage tracing and intravital imaging supports the presence of a transient population of migratory adipocytes. After participating in innate defense and metabolic support, these cells appear to gradually exit the wound environment through lipolytic activity and migration rather than stable cross-lineage transdifferentiation (17). Such a pattern may facilitate the orderly turnover of cell populations at the wound repair front. The relative contribution of these distinct cellular trajectories may depend on the wound context and remains to be fully defined.

**Figure 4:**
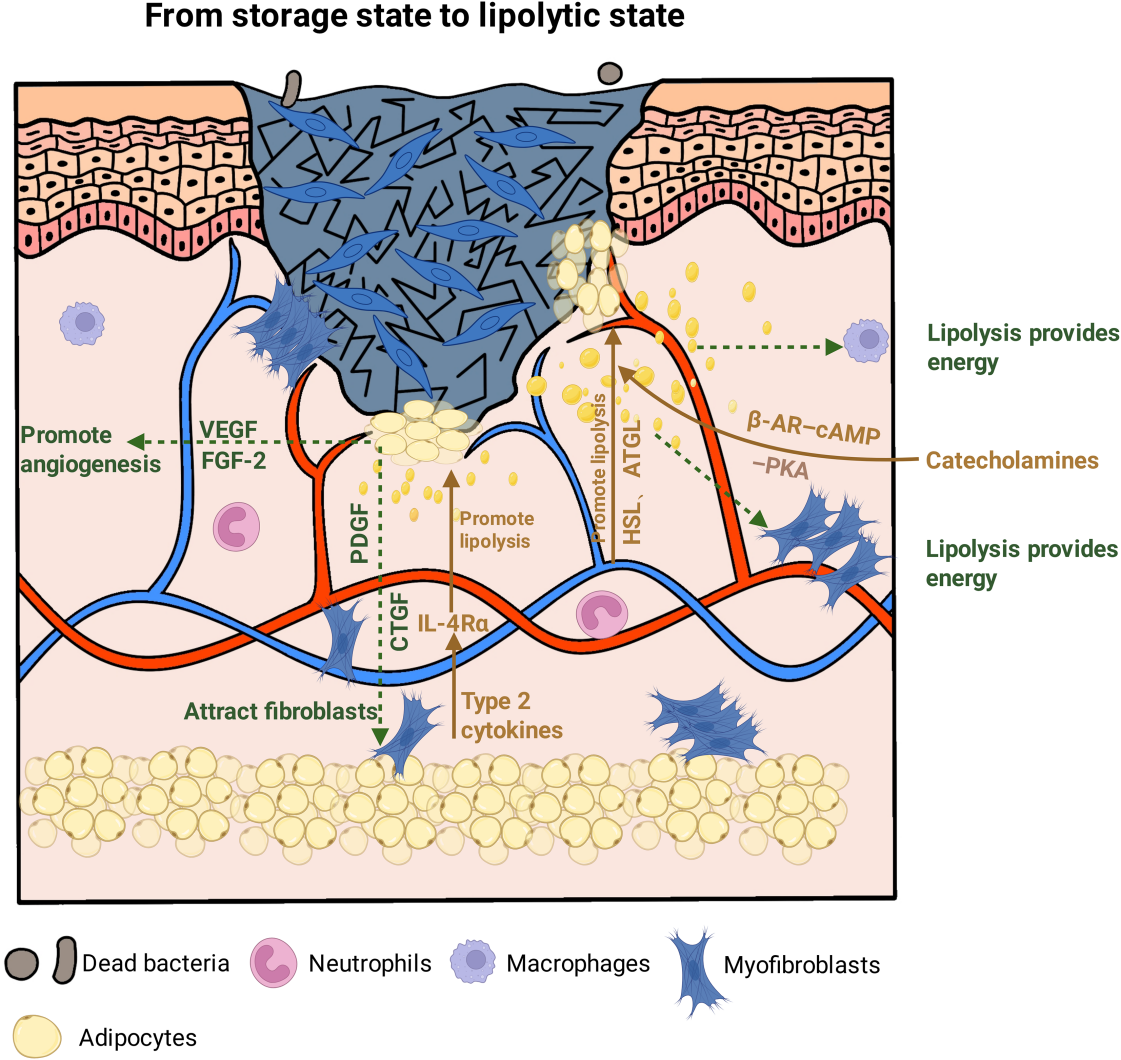
Lipolysis-driven regression of the adipose barrier during wound repair. During the proliferative phase of wound healing, adipocytes undergo enhanced lipolysis and reduced lipid storage. This process is regulated by catecholamine–β-adrenergic receptor–cAMP–PKA signaling and type 2 cytokine–IL-4Rα–PKA–HSL pathways, leading to triglyceride hydrolysis by ATGL and HSL and the release of metabolic substrates into the wound microenvironment. In parallel, adipocytes secrete pro-angiogenic and pro-fibrotic mediators, including VEGF, FGF-2, and PDGF, supporting angiogenesis, fibroblast recruitment, granulation tissue formation, and re-epithelialization.

Regression of the adipose barrier is influenced by multiple upstream signaling inputs. Type-2 cytokines enhance dermal adipocyte lipolysis through the IL-4Rα–PKA–HSL signaling pathway (74, 75); concurrently, catecholamines operate via the canonical β-AR–cAMP–PKA axis, working cooperatively to promote triglyceride hydrolysis mediated by HSL and ATGL (76). Functional studies show that suppression of ATGL delays wound repair, underscoring the essential role of lipolytic pathways in coordinating the timing of tissue regeneration (25). The lipolysis-driven regression of the adipose barrier during wound repair is illustrated in **Figure 4**.

### 3.2 Co-initiation of energy switching and tissue remodeling

The regression of the adipose barrier is accompanied by marked changes in adipocyte function. As wound repair progresses, adipocytes shift from early defense-associated activities toward processes that support later stages of tissue reconstruction through multiple mechanisms.

#### 3.2.1 Regulation of fibroblast activity and extracellular matrix remodeling

Adipocytes and fibroblasts, which arise from a shared mesenchymal lineage, interact closely during the reparative phase of wound healing (77, 78). Through the secretion of paracrine factors such as platelet-derived growth factor and connective tissue growth factor, adipocytes promote recruitment of fibroblasts to the wound site and modulation of their activation state (15, 79). Experimental studies have shown that selective depletion of adipocytes within the injured area is accompanied by a marked reduction in fibroblast infiltration and delayed wound closure. By contrast, the presence of adipocytes is associated with more organized extracellular matrix deposition and a lower propensity for excessive scar formation (79). In the absence of adipocytes, fibroblasts often remain in a persistently activated state and produce excessive amounts of collagen and other extracellular matrix components, leading to progressive tissue stiffening and scar formation (80). As matrix rigidity increases, mechanical cues transmitted through pathways such as integrin–FAK–ROCK interfere with the differentiation of adipocyte precursors (adipose-derived stem cells) and limit local adipose regeneration. Impaired adipose recovery, in turn, removes an important regulatory constraint on fibroblast activity, allowing matrix deposition and stiffening to continue. Through this sequence of events, loss of adipose tissue, fibroblast overactivation, and matrix stiffening become mutually reinforcing, promoting a self-sustaining fibrotic environment (81). Evidence from regenerative biology indicates that cell fate at the wound site is not irrevocably fixed. Notably, microenvironmental cues associated with newly formed hair follicles have been shown to promote the conversion of differentiated myofibroblasts toward adipocyte-like phenotypes (82, 83). Such observations point to the possibility that manipulating local signaling conditions during late stages of repair may influence fibroblast plasticity and limit fibrotic remodeling, thereby facilitating restoration of normal dermal architecture.

#### 3.2.2 Promoting angiogenesis to secure vascular supply for repair

Adequate blood supply is essential for tissue reconstruction (84–86). During the proliferative phase of wound healing, mature adipocytes and adipose-derived stem or stromal cells promote angiogenesis by releasing pro-angiogenic factors like vascular endothelial growth factor (VEGF) and fibroblast growth factor 2 (FGF-2). These cells also provide metabolic substrates that support endothelial growth, which is linked to increased capillary density and improved granulation tissue maturation (87, 88). In the hypoxic environment typically found in infected wounds, adipose stromal cells display heightened angiogenic activity, likely as an adaptive response to limited oxygen availability (89). Additionally, the developing vascular network is crucial for the survival and functional recovery of adipocytes. The interactions between adipose cells and the wound vasculature play a key role in determining the efficiency and quality of tissue repair (90).

#### 3.2.3 Accelerating re-epithelialization to achieve wound closure

Restoration of an intact epidermal barrier represents a key endpoint of successful wound repair (91). Adipocyte-derived leptin enhances keratinocyte proliferation and migratory capacity (92). Additionally, the antimicrobial peptide LL-37, which plays an important role during early host defense, also exerts regulatory functions during the proliferative phase of healing. LL-37 acts as a chemotactic signal for epidermal cells and stimulates their production of growth factors, processes that are associated with the progression of re-epithelialization through multiple pathways (93). This supports the timely re-establishment of the epidermal barrier once the infectious challenge has been controlled.

### 3.3 Global switching of the reparative microenvironment and determination of scar outcomes

Following regression of the adipose barrier after its early contributions to host defense, the dominant regulatory processes within the wound environment shift from inflammation driven by immune cell activity toward mechanisms governing matrix regulation. This later phase is characterized by increased involvement of TGF-β signaling and mechanical forces within the tissue. Such changes are thought to influence the trajectory of subsequent repair outcomes.

TGF-β, particularly the TGF-β1 isoform, signals through the cell surface TβRII and TβRI receptor complex to activate downstream Smad2 and Smad3. Activation of this pathway is associated with increased expression of genes encoding extracellular matrix components, including type I collagen and fibronectin, and with the differentiation of fibroblasts toward a contractile myofibroblast phenotype (94, 95). In parallel, mechanical forces generated during wound healing activate cytoskeletal organization and contractile signaling through integrin-dependent focal adhesion kinase and RhoA-associated kinase pathways. These mechanotransduction processes have been shown to promote myofibroblast activation and to interact with TGF-β–Smad signaling, contributing to enhanced extracellular matrix deposition and remodeling (96, 97). Such combined biochemical and mechanical inputs are thought to influence the maturation of granulation tissue during repair. The activity of these pathways is further modulated by local microenvironmental conditions. For example, at hypoxic and mechanically stressed wound margins, the tetraspanin CD9 has been reported to facilitate assembly of the TβRII–TβRI receptor complex, increasing cellular responsiveness to TGF-β signaling and favoring fibrotic outcomes (98); conversely, experimental reduction of mechanical tension or inhibition of downstream effectors such as YAP and TAZ has been associated with attenuated scar formation (99).

The timely regression of the adipose barrier represents a critical transition during wound repair. As adipocytes undergo lipolysis, they release metabolic substrates and signaling mediators that support tissue reconstruction while creating space within the wound bed for the expansion and activity of fibroblasts, endothelial cells, and epithelial cells. Concurrently, matrix-regulating processes dominated by transforming growth factor-β signaling and mechanical tension become increasingly prominent, governing later stages of repair. Although adipocytes withdraw from the wound front, their continued metabolic activity, fatty acid release, and paracrine signaling persistently influence the wound microenvironment and modulate extracellular matrix remodeling. Together, these interconnected processes shape the trajectory of healing, determining outcomes that range from effective tissue regeneration to fibrotic scar formation with residual functional impairment. A deeper understanding of this dynamic transition may inform the development of therapeutic strategies for chronic, non-healing wounds and pathological scarring.

## 4 Clinical translation and future outlook: from mechanism to application

Clinical studies of adipose-based interventions for infected or hard-to-heal wounds are summarized in **Table 3**.

**Table 3:** Clinical studies of adipose-based interventions for infected or hard-to-heal wounds.

Year	First author	Wound / population	Design (n)	Intervention	Comparator	Main findings
2018	Didangelos	refractory diabetic foot ulcer	Case report (1)	autologous SVF + PRP applied to wound	——	healed within 1 month; remained closed 2 years
2021	Carstens	chronic DFU (>3 cm), T2D	Phase I (63)	30×10^6 autologous SVF cells injected (ulcer bed/periphery + pedal arteries)	——	no intervention-related SAEs; 6 mo: 51 complete closure; 12 mo: 50 complete closure
2021	Tanios	chronic ulcers	RCT (100)	debridement + autologous SVF injection every 3 weeks	debridement + conventional dressing	study group showed improved healing
2021	Dung	chronic wounds	pilot longitudinal (30 pts / 38 wounds)	autologous ADSC sheet grafting	——	reported structural/ultrastructural improvement over 3-week follow-up
2022	Abouzaid	superficial/deep dermal burns	RCT (100; 50/50)	AFG injection + nanofat dressing	conventional topical care	shorter hospital stay; less skin grafting; fewer contractures; improved scar texture
2023	Mrozikiewicz-Rakowska	diabetic foot ulcers	RCT (46; 23/23)	allogenic ADSCs in fibrin gel + SOC	SOC	greater wound reduction; day 49: 7 healed vs 1; no severe AEs

### 4.1 Prioritizing key regulators to target adipocyte function and promote wound healing

Advances in understanding adipocyte roles in wound repair—including antimicrobial peptide production, immune cell interactions, and angiogenic regulation—have highlighted adipocyte signaling pathways as promising therapeutic targets. Key signaling pathways involved in adipocyte-mediated responses during infected wound repair are summarized in **Table 4**. Strategies using small molecules, biologics, or gene-editing technologies could selectively enhance adipocyte functions, including antimicrobial, immunomodulatory, and pro-regenerative activities, as well as adipocyte migration, differentiation, and paracrine signaling (100). Instead of introducing exogenous cells, these approaches focus on regulating endogenous adipocytes at the molecular level. This method offers advantages in terms of practicality and controllability and could complement existing wound healing therapies aimed at improving closure.

**Table 4:** Key signaling pathways involved in adipocyte-mediated responses during infected wound.

Signaling pathway	Principal function(s) in infected wounds	Proximal activators / ligands	Representative studies
Cdc42/Rac1–Arp2/3 pathway	Drives directed adipocyte migration and morphological remodeling	Injury- and infection-related cues (e.g., PDGF, TGF-β)	Andrieu et al. (2025) (20)
RhoA–ROCK–myosin II pathway	Promotes adipocyte motility and regulates cytoskeletal reorganization	Damage-associated signals (e.g., PDGF, TGF-β)	Andrieu et al. (2025) (20)
TLR2/TLR4	Activates adipocyte production of AMPs; augments innate immune responses	Bacterial/fungal PAMPs (e.g., lipoproteins, peptidoglycan, LPS)	Hopfinger et al. (2021) (38)
C3a/C3b (complement) pathway	Modulates immune responses via the complement cascade; enhances pathogen clearance	adipsin	Ma et al. (2024); Luo et al. (2024) (47, 51)

### 4.2 Autologous fat grafting as a reparative strategy in infected wounds

Autologous fat grafting has a long-standing history in plastic and reconstructive surgery and has more recently been explored for scar modulation and the management of chronic, infected wounds (101, 102). Clinically, adipose tissue is most often harvested from donor sites such as the abdomen or thigh, processed, and then placed directly into the wound bed. Rather than acting solely as a space-filling material, grafted fat delivers a heterogeneous population of cells and stromal components, including adipose-derived stem cells, elements of the stromal vascular fraction, and mature adipocytes. Accumulating experimental and clinical evidence suggests that these components contribute to multiple aspects of tissue repair, extending the role of fat grafting beyond structural support alone (103–105).

Autologous fat grafting offers several practical advantages in clinical settings. Because the transplanted tissue is autologous, the risk of immune rejection is minimal, and adipose tissue can usually be obtained in sufficient quantities, allowing procedures to be repeated when necessary (106). Despite these strengths, the clinical outcome of fat grafting is often limited by variable graft survival, a problem that becomes particularly evident in poorly vascularized or infected wound beds (107). To address this limitation, a range of optimization strategies has been explored. These include refinements in harvesting and processing techniques to preserve cell viability, as well as combination approaches in which fat grafts are supplemented with bioactive additives such as platelet-rich plasma, enriched stromal vascular fractions, or extracellular vesicles to enhance paracrine support (108, 109). In parallel, biomaterial-based approaches, including injectable hydrogels and scaffold systems, have been developed to provide mechanical support and to create a local microenvironment conducive to sustained growth factor activity (110). Taken together, these developments suggest that autologous fat grafting may serve as a useful adjunct within broader therapeutic strategies for the management of infected wounds, while continued optimization is required to address its current limitations.

### 4.3 Gene-edited porcine adipocytes and tissue-engineered products: opening a new xenogeneic therapeutic source

To overcome limitations associated with insufficient autologous adipose tissue in large wounds, as well as the need for standardized and readily available therapeutic materials, xenogeneic adipose tissue-based engineered approaches have attracted increasing attention. Advances in gene-edited porcine models developed for xenotransplantation have provided experimental evidence suggesting the feasibility of modifying porcine adipose cells for biomedical applications and have outlined potential technical frameworks for their further investigation (111, 112).

Advances in CRISPR-Cas9-based genome editing have enabled targeted modification of porcine adipocytes, including the disruption of major xenoantigen encoding genes such as α-1, 3-galactosyltransferase, as well as the introduction of selected human immune regulatory genes, for example CD47 and HLA-E (113). These genetic modifications have been explored as strategies to attenuate immunogenic responses associated with xenogeneic cells. Engineered porcine adipocytes with such humanized features have been proposed to exhibit improved immune compatibility in preclinical experimental settings, while retaining key adipocyte-associated functions, including antimicrobial peptide secretion, paracrine signaling, and support of angiogenic processes.

At the level of tissue engineering, porcine adipocytes have been explored in combination with a range of natural or synthetic scaffold materials, including collagen-based hydrogels, decellularized dermal matrices, and biodegradable polymer frameworks (114–116), to create bioactive skin substitutes. Such products are being explored as readily available therapeutic materials, particularly in clinical settings where extensive burns or complex infected wounds are accompanied by limited autologous donor tissue, a situation that can otherwise constrain timely intervention.

Several key challenges remain to be addressed, including the potential transmission of xenogeneic pathogens such as porcine endogenous retroviruses (117), the scalability of manufacturing processes with standardized quality control, and the clarification of ethical and regulatory frameworks governing clinical use. At the same time, ongoing efforts that integrate genome editing with tissue engineering approaches are expanding the experimental basis for porcine-adipocyte based products and informing future translational research, although substantial evaluation is still required before clinical implementation can be considered.

### 4.4 Spatial omics and multi-omics strategies to bridge mechanism and translation in infected wound therapies

Spatial omics as an enabling strategy for translation. Infected wounds are highly heterogeneous in space, spanning distinct microanatomical niches (wound edge, granulation tissue, and dermal adipose) that cannot be fully resolved by bulk profiling. Spatially resolved and multimodal approaches (e.g., spatial transcriptomics and high-plex spatial protein imaging; such as Xenium and PhenoCycler-Fusion 2.0) can therefore help bridge mechanism and translation by assigning the cell-of-origin of key mediators, quantifying immune–adipose neighborhood architecture, and prioritizing actionable pathways for functional validation. Recent studies have demonstrated that integrating single-cell and spatial transcriptomic readouts can localize cell states within healing wounds and reveal spatially organized stromal–immune programs, providing a blueprint for extending similar analyses to infected wound settings (118). In addition, multiplex spatial profiling technologies have been highlighted as complementary tools to map immune phenotypes and tissue organization *in situ*, which may support response biomarker development and mechanism-informed evaluation of adipose-based therapies and engineered products (119).

## 5 Conclusion

This review summarizes current evidence highlighting the diverse roles of adipocytes in infected wound repair. Beyond their classical metabolic functions, adipocytes participate in multiple aspects of the healing process, including antimicrobial defense, regulation of immune responses, and support of tissue regeneration and remodeling. Through interactions with immune cells, stromal cells, and the extracellular matrix, adipocytes contribute to the dynamic regulation of the wound microenvironment. Collectively, these findings broaden current understanding of adipocyte biology in the context of tissue injury and infection. They further suggest that therapeutic strategies targeting the local wound microenvironment, including adipocyte-associated pathways, may complement existing approaches to infection control and tissue repair, although further investigation is required to translate these insights into clinically effective interventions.

## References

[1] Martin P Nunan R . Cellular and molecular mechanisms of repair in acute and chronic wound healing. Br J Dermatol. (2015) 173:370–8. doi: 10.1111/bjd.13954, PMID: 26175283 PMC4671308

[2] Wilkinson HN Hardman MJ . Wound healing: cellular mechanisms and pathological outcomes. Open Biol. (2020) 10:200223. doi: 10.1098/rsob.200223, PMID: 32993416 PMC7536089

[3] Sen CK . Human wounds and its burden: an updated compendium of estimates. Adv Wound Care (New Rochelle). (2019) 8:39–48. doi: 10.1089/wound.2019.0946, PMID: 30809421 PMC6389759

[4] Carter MJ DaVanzo J Haught R Nusgart M Cartwright D Fife CE . Chronic wound prevalence and the associated cost of treatment in medicare beneficiaries: changes between 2014 and 2019. J Med Econ. (2023) 26:894–901. doi: 10.1080/13696998.2023.2232256, PMID: 37415496

[5] Swanson T Ousey K Haesler E Bjarnsholt T Carville K Idensohn P . IWII Wound Infection in Clinical Practice consensus document: 2022 update. J Wound Care. (2022) 31:S10–21. doi: 10.12968/jowc.2022.31.Sup12.S10, PMID: 36475844

[6] Diban F Di Lodovico S Di Fermo P D’Ercole S D’Arcangelo S Di Giulio M . Biofilms in chronic wound infections: innovative antimicrobial approaches using the *in vitro* lubbock chronic wound biofilm model. Int J Mol Sci. (2023) 24(2):1004. doi: 10.3390/ijms24021004, PMID: 36674518 PMC9862456

[7] Liu X Chu H Ji Y Bosnjak Z Ao H Li T . Which bmi for diabetes patients is better? From the view of the adipose tissue macrophage-derived exosome. Diabetes Metab Syndr Obes. (2022) 15:141–53. doi: 10.2147/DMSO.S345890, PMID: 35046685 PMC8763208

[8] Cinti S . The adipose organ at a glance. Dis Model Mech. (2012) 5:588–94. doi: 10.1242/dmm.009662, PMID: 22915020 PMC3424455

[9] Sakers A De Siqueira MK Seale P Villanueva CJ . Adipose-tissue plasticity in health and disease. Cell. (2022) 185:419–46. doi: 10.1016/j.cell.2021.12.016, PMID: 35120662 PMC11152570

[10] Cerk IK Wechselberger L Oberer M . Adipose triglyceride lipase regulation: an overview. Curr Protein Pept Sci. (2018) 19:221–33. doi: 10.2174/1389203718666170918160110, PMID: 28925902 PMC7613786

[11] Choe SS Huh JY Hwang IJ Kim JI Kim JB . Adipose tissue remodeling: its role in energy metabolism and metabolic disorders. Front Endocrinol (Lausanne). (2016) 7:30. doi: 10.3389/fendo.2016.00030, PMID: 27148161 PMC4829583

[12] Alexander CM Kasza I Yen CL Reeder SB Hernando D Gallo RL . Dermal white adipose tissue: A new component of the thermogenic response. J Lipid Res. (2015) 56:2061–9. doi: 10.1194/jlr.R062893, PMID: 26405076 PMC4617393

[13] Zhang LJ Guerrero-Juarez CF Hata T Bapat SP Ramos R Plikus MV . Innate immunity. Dermal adipocytes protect against invasive Staphylococcus aureus skin infection. Science. (2015) 347:67–71. doi: 10.1126/science.1260972, PMID: 25554785 PMC4318537

[14] Franz A Wood W Martin P . Fat body cells are motile and actively migrate to wounds to drive repair and prevent infection. Dev Cell. (2018) 44:460–70.e3. doi: 10.1016/j.devcel.2018.01.026, PMID: 29486196 PMC6113741

[15] Eming SA Martin P Tomic-Canic M . Wound repair and regeneration: mechanisms, signaling, and translation. Sci Transl Med. (2014) 6:265sr6. doi: 10.1126/scitranslmed.3009337, PMID: 25473038 PMC4973620

[16] Kim J Park KY Choi S Ko UH Lim DS Suh JM . Ceiling culture chip reveals dynamic lipid droplet transport during adipocyte dedifferentiation via actin remodeling. Lab Chip. (2022) 22:3920–32. doi: 10.1039/d2lc00428c, PMID: 36097851

[17] Kalgudde Gopal S Dai R Stefanska AM Ansari M Zhao J Ramesh P . Wound infiltrating adipocytes are not myofibroblasts. Nat Commun. (2023) 14:3020. doi: 10.1038/s41467-023-38591-6, PMID: 37230982 PMC10213017

[18] Yao L Jeong S Kwon HR Olson LE . Regulation of adipocyte dedifferentiation at the skin wound edge. bioRxiv. (2023). doi: 10.1101/2023.11.22.568302, PMID: 38045303 PMC10690246

[19] Lucas T Waisman A Ranjan R Roes J Krieg T Muller W . Differential roles of macrophages in diverse phases of skin repair. J Immunol. (2010) 184:3964–77. doi: 10.4049/jimmunol.0903356, PMID: 20176743

[20] Andrieu C Hunyi Lee B Franz A . Cell deformations generated by stochastic actomyosin waves drive *in vivo* random-walk swimming migration. J Cell Sci. (2025) 138(10):jcs263787. doi: 10.1242/jcs.263787, PMID: 40183280 PMC12148041

[21] George A Martin P . Wound healing insights from flies and fish. Cold Spring Harb Perspect Biol. (2022) 14(11):a041217. doi: 10.1101/cshperspect.a041217, PMID: 35817511 PMC9620851

[22] Stadelmann WK Digenis AG Tobin GR . Physiology and healing dynamics of chronic cutaneous wounds. Am J Surg. (1998) 176:26S–38S. doi: 10.1016/s0002-9610(98)00183-4, PMID: 9777970

[23] Zhang Z Shao M Hepler C Zi Z Zhao S An YA . Dermal adipose tissue has high plasticity and undergoes reversible dedifferentiation in mice. J Clin Invest. (2019) 129:5327–42. doi: 10.1172/JCI130239, PMID: 31503545 PMC6877341

[24] Li Y Long J Zhang Z Yin W . Insights into the unique roles of dermal white adipose tissue (Dwat) in wound healing. Front Physiol. (2024) 15:1346612. doi: 10.3389/fphys.2024.1346612, PMID: 38465261 PMC10920283

[25] Shook BA Wasko RR Mano O Rutenberg-Schoenberg M Rudolph MC Zirak B . Dermal adipocyte lipolysis and myofibroblast conversion are required for efficient skin repair. Cell Stem Cell. (2020) 26:880–95 e6. doi: 10.1016/j.stem.2020.03.013, PMID: 32302523 PMC7853423

[26] Abyzov A Vaccarino FM . Cell lineage tracing and cellular diversity in humans. Annu Rev Genomics Hum Genet. (2020) 21:101–16. doi: 10.1146/annurev-genom-083118-015241, PMID: 32413272

[27] Trapnell C Cacchiarelli D Grimsby J Pokharel P Li S Morse M . The dynamics and regulators of cell fate decisions are revealed by pseudotemporal ordering of single cells. Nat Biotechnol. (2014) 32:381–6. doi: 10.1038/nbt.2859, PMID: 24658644 PMC4122333

[28] Casano AM Sixt M . A fat lot of good for wound healing. Dev Cell. (2018) 44:405–6. doi: 10.1016/j.devcel.2018.02.009, PMID: 29486189

[29] Adib Y Bensussan A Michel L . Cutaneous wound healing: A review about innate immune response and current therapeutic applications. Mediators Inflammation. (2022) 2022:5344085. doi: 10.1155/2022/5344085, PMID: 35509434 PMC9061066

[30] Tan ST Dosan R . Lessons from epithelialization: the reason behind moist wound environment. Open Dermatol J. (2019) 13:34–40. doi: 10.2174/1874372201913010034, PMID: 41727521

[31] Wilgus TA Roy S McDaniel JC . Neutrophils and wound repair: positive actions and negative reactions. Adv Wound Care. (2013) 2:379–88. doi: 10.1089/wound.2012.0383, PMID: 24527354 PMC3763227

[32] Koh TJ DiPietro LA . Inflammation and wound healing: the role of the macrophage. Expert Rev Mol Med. (2011) 13:e23. doi: 10.1017/s1462399411001943, PMID: 21740602 PMC3596046

[33] Braff MH Zaiou M Fierer J Nizet V Gallo RL . Keratinocyte production of cathelicidin provides direct activity against bacterial skin pathogens. Infect Immun. (2005) 73:6771–81. doi: 10.1128/IAI.73.10.6771-6781.2005, PMID: 16177355 PMC1230954

[34] Hohl TM Wang J Duan Z Zeng R Yang L Liu W . Antimicrobial peptide-producing dermal preadipocytes defend against candida albicans skin infection via the fgfr-mek-erk pathway. PloS Pathog. (2023) 19(11):e1011754. doi: 10.1371/journal.ppat.1011754, PMID: 38032898 PMC10688742

[35] Veenstra KA Wangkahart E Wang T Tubbs L Ben Arous J Secombes CJ . Rainbow trout (Oncorhynchus mykiss) adipose tissue undergoes major changes in immune gene expression following bacterial infection or stimulation with pro-inflammatory molecules. Dev Comp Immunol. (2018) 81:83–94. doi: 10.1016/j.dci.2017.11.001, PMID: 29126991

[36] Adnan SB Maarof M Fauzi MB Md Fadilah NI . Antimicrobial peptides in wound healing and skin regeneration: dual roles in immunity and microbial defense. Int J Mol Sci. (2025) 26(13):5920. doi: 10.3390/ijms26135920, PMID: 40649700 PMC12249706

[37] Hopfinger A Schmid A Schweitzer L Patz M Weber A Schaffler A . Regulation of cathelicidin antimicrobial peptide (Camp) gene expression by tnfalpha and cfdna in adipocytes. Int J Mol Sci. (2023) 24(21):15820. doi: 10.3390/ijms242115820, PMID: 37958808 PMC10649744

[38] Hopfinger A Karrasch T Schaffler A Schmid A . Regulation of camp (Cathelicidin antimicrobial peptide) expression in adipocytes by tlr 2 and 4. Innate Immun. (2021) 27:184–91. doi: 10.1177/1753425920988167, PMID: 33509002 PMC7882808

[39] Gombart AF Borregaard N Koeffler HP . Human cathelicidin antimicrobial peptide (Camp) gene is a direct target of the vitamin D receptor and is strongly up-regulated in myeloid cells by 1, 25-dihydroxyvitamin D3. FASEB J. (2005) 19:1067–77. doi: 10.1096/fj.04-3284com, PMID: 15985530

[40] Zielke C Nielsen JE Lin JS Barron AE . Between good and evil: complexation of the human cathelicidin ll-37 with nucleic acids. Biophys J. (2024) 123:1316–28. doi: 10.1016/j.bpj.2023.10.035, PMID: 37919905 PMC11163296

[41] Su Y Sharma NS John JV Ganguli-Indra G Indra AK Gombart AF . Engineered exosomes containing cathelicidin/ll-37 exhibit multiple biological functions. Adv Healthc Mater. (2022) 11:e2200849. doi: 10.1002/adhm.202200849, PMID: 35930707 PMC9588668

[42] Martinez-Santibañez G Cho KW Lumeng CN . Imaging white adipose tissue with confocal microscopy. Methods Adipose Tissue Biology Part A. Methods Enzymology. (2014) 537:17–30. doi: 10.1016/B978-0-12-411619-1.00002-1, PMID: 24480339 PMC4233125

[43] Takahashi M Umehara Y Yue H Trujillo-Paez JV Peng G Nguyen HLT . The antimicrobial peptide human beta-defensin-3 accelerates wound healing by promoting angiogenesis, cell migration, and proliferation through the fgfr/jak2/stat3 signaling pathway. Front Immunol. (2021) 12:712781. doi: 10.3389/fimmu.2021.712781, PMID: 34594328 PMC8476922

[44] Wu Z Wang Z Chen T Wang D Zhou F Zhang G . Dermal white adipose tissue: A new modulator in wound healing and regeneration. Regener Ther. (2025) 28:115–25. doi: 10.1016/j.reth.2024.11.015, PMID: 39717110 PMC11665542

[45] Kobayashi T Nagao K . Deepening” Insight on skin aging and anti-microbial immunity. Cell Metab. (2019) 29:515–7. doi: 10.1016/j.cmet.2019.02.006, PMID: 30840910

[46] Kong Y Wang N Tong Z Wang D Wang P Yang Q . Role of complement factor D in cardiovascular and metabolic diseases. Front Immunol. (2024) 15:1453030. doi: 10.3389/fimmu.2024.1453030, PMID: 39416783 PMC11479899

[47] King BC Blom AM . Intracellular complement and immunometabolism: the advantages of compartmentalization. Eur J Immunol. (2024) 54:e2350813. doi: 10.1002/eji.202350813, PMID: 38757569

[48] Luo P Xin W Guo S Li X Zhang Q Xu Y . Structural insights into the agonist activity of the nonpeptide modulator jr14a on C3ar. Cell Discov. (2025) 11:7. doi: 10.1038/s41421-024-00765-x, PMID: 39788969 PMC11718183

[49] Boero E Gorham RD Jr. Francis EA Brand J Teng LH Doorduijn DJ . Purified complement C3b triggers phagocytosis and activation of human neutrophils via complement receptor 1. Sci Rep. (2023) 13:274. doi: 10.1038/s41598-022-27279-4, PMID: 36609665 PMC9822988

[50] Yoshida J Hayashi T Munetsuna E Khaledian B Sueishi F Mizuno M . Adipsin-dependent adipocyte maturation induces cancer cell invasion in breast cancer. Sci Rep. (2024) 14:18494. doi: 10.1038/s41598-024-69476-3, PMID: 39122742 PMC11316094

[51] Ma L Gilani A Rubio-Navarro A Cortada E Li A Reilly SM . Adipsin and adipocyte-derived C3ar1 regulate thermogenic fat in a sex-dependent fashion. JCI Insight (2024) 9(11):e178925. doi: 10.1172/jci.insight.178925, PMID: 38713526 PMC11382875

[52] Rosen BS Cook KS Yaglom J Groves DL Volanakis JE Damm D . Adipsin and complement factor D activity: an immune-related defect in obesity. Science. (1989) 244:1483–7. doi: 10.1126/science.2734615, PMID: 2734615

[53] Cazander G Jukema GN Nibbering PH . Complement activation and inhibition in wound healing. Clin Dev Immunol. (2012) 2012:534291. doi: 10.1155/2012/534291, PMID: 23346185 PMC3546472

[54] Yoon G Puentes R Tran J Multani A Cobo ER . The role of cathelicidins in neutrophil biology. J Leukoc Biol. (2024) 116:689–705. doi: 10.1093/jleuko/qiae112, PMID: 38758953

[55] Bruun JM Lihn AS Madan AK Pedersen SB Schiøtt KM Fain JN . Higher production of IL-8 in visceral vs. subcutaneous adipose tissue. Implication of nonadipose cells in adipose tissue. Am J Physiol Endocrinol Metab. (2004) 286:E8–13. doi: 10.1152/ajpendo.00269.2003, PMID: 13129857

[56] Cambier S Gouwy M Proost P . The chemokines cxcl8 and cxcl12: molecular and functional properties, role in disease and efforts towards pharmacological intervention. Cell Mol Immunol. (2023) 20:217–51. doi: 10.1038/s41423-023-00974-6, PMID: 36725964 PMC9890491

[57] Zhu Z Zhou S Li S Gong S Zhang Q . Neutrophil extracellular traps in wound healing. Trends Pharmacol Sci. (2024) 45:1033–45. doi: 10.1016/j.tips.2024.09.007, PMID: 39419742

[58] Wang H Kim SJ Lei Y Wang S Wang H Huang H . Neutrophil extracellular traps in homeostasis and disease. Signal Transduct Target Ther. (2024) 9:235. doi: 10.1038/s41392-024-01933-x, PMID: 39300084 PMC11415080

[59] Zeng X Wang TW Yamaguchi K Hatakeyama S Yamazaki S Shimizu E . M2 macrophage-derived tgf-beta induces age-associated loss of adipogenesis through progenitor cell senescence. Mol Metab. (2024) 84:101943. doi: 10.1016/j.molmet.2024.101943, PMID: 38657734 PMC11079528

[60] Sohn JH Lee YK Han JS Jeon YG Kim JI Choe SS . Perilipin 1 (Plin1) deficiency promotes inflammatory responses in lean adipose tissue through lipid dysregulation. J Biol Chem. (2018) 293:13974–88. doi: 10.1074/jbc.RA118.003541, PMID: 30042231 PMC6130955

[61] Sakai S Sato K Tabata Y Kishi K . Local release of pioglitazone (a peroxisome proliferator-activated receptor gamma agonist) accelerates proliferation and remodeling phases of wound healing. Wound Repair Regener. (2016) 24:57–64. doi: 10.1111/wrr.12376, PMID: 26710090

[62] Takemura Y Ouchi N Shibata R Aprahamian T Kirber MT Summer RS . Adiponectin modulates inflammatory reactions via calreticulin receptor-dependent clearance of early apoptotic bodies. J Clin Invest. (2007) 117:375–86. doi: 10.1172/JCI29709, PMID: 17256056 PMC1770947

[63] Zhu D Johnson TK Wang Y Thomas M Huynh K Yang Q . Macrophage M2 polarization induced by exosomes from adipose-derived stem cells contributes to the exosomal proangiogenic effect on mouse ischemic hindlimb. Stem Cell Res Ther. (2020) 11:162. doi: 10.1186/s13287-020-01669-9, PMID: 32321589 PMC7178595

[64] Yin D Shen G . Exosomes from adipose-derived stem cells regulate macrophage polarization and accelerate diabetic wound healing via the circ-rps5/mir-124-3p axis. Immun Inflammation Dis. (2024) 12:e1274. doi: 10.1002/iid3.1274, PMID: 38888351 PMC11184652

[65] Kuninaka Y Ishida Y Ishigami A Nosaka M Matsuki J Yasuda H . Macrophage polarity and wound age determination. Sci Rep. (2022) 12:20327. doi: 10.1038/s41598-022-24577-9, PMID: 36434083 PMC9700750

[66] Han MS White A Perry RJ Camporez JP Hidalgo J Shulman GI . Regulation of adipose tissue inflammation by interleukin 6. Proc Natl Acad Sci U.S.A. (2020) 117:2751–60. doi: 10.1073/pnas.1920004117, PMID: 31980524 PMC7022151

[67] Zhang Y Zhang B Sun X . The molecular mechanism of macrophage-adipocyte crosstalk in maintaining energy homeostasis. Front Immunol. (2024) 15:1378202. doi: 10.3389/fimmu.2024.1378202, PMID: 38650945 PMC11033412

[68] Zhou X Guo YL Xu C Wang J . Macrophages: key players in diabetic wound healing. World J Diabetes. (2024) 15:2177–81. doi: 10.4239/wjd.v15.i11.2177, PMID: 39582557 PMC11580577

[69] Liao X Zeng Q Xie L Zhang H Hu W Xiao L . Adipose stem cells control obesity-induced T cell infiltration into adipose tissue. Cell Rep. (2024) 43:113963. doi: 10.1016/j.celrep.2024.113963, PMID: 38492218

[70] Satoh M Hoshino M Fujita K Iizuka M Fujii S Clingan CS . Adipocyte-specific cd1d-deficiency mitigates diet-induced obesity and insulin resistance in mice. Sci Rep. (2016) 6:28473. doi: 10.1038/srep28473, PMID: 27329323 PMC4916414

[71] van Eijkeren RJ Krabbe O Boes M Schipper HS Kalkhoven E . Endogenous lipid antigens for invariant natural killer T cells hold the reins in adipose tissue homeostasis. Immunology. (2018) 153:179–89. doi: 10.1111/imm.12839, PMID: 28898395 PMC5765379

[72] Huh JY Park J Kim JI Park YJ Lee YK Kim JB . Deletion of CD1d in adipocytes aggravates adipose tissue inflammation and insulin resistance in obesity. Diabetes. (2017) 66:835–47. doi: 10.2337/db16-1122, PMID: 28082459

[73] Marangoni RG Korman BD Wei J Wood TA Graham LV Whitfield ML . Myofibroblasts in murine cutaneous fibrosis originate from adiponectin-positive intradermal progenitors. Arthritis Rheumatol. (2015) 67:1062–73. doi: 10.1002/art.38990, PMID: 25504959 PMC4472310

[74] Satzinger S Willenborg S Ding X Klose CSN Radtke D Voehringer D . Type 2 immunity regulates dermal white adipose tissue function. J Invest Dermatol. (2023) 143:2456–67 e5. doi: 10.1016/j.jid.2023.05.017, PMID: 37295491

[75] Shiau MY Chuang PH Yang CP Hsiao CW Chang SW Chang KY . Mechanism of interleukin-4 reducing lipid deposit by regulating hormone-sensitive lipase. Sci Rep. (2019) 9:11974. doi: 10.1038/s41598-019-47908-9, PMID: 31427606 PMC6700157

[76] Zhu JY Guo L . Exercise-regulated lipolysis: its role and mechanism in health and diseases. J Adv Res. (2025) 75:291–309. doi: 10.1016/j.jare.2024.11.031, PMID: 39613256 PMC12536597

[77] Joe AW Yi L Natarajan A Le Grand F So L Wang J . Muscle injury activates resident fibro/adipogenic progenitors that facilitate myogenesis. Nat Cell Biol. (2010) 12:153–63. doi: 10.1038/ncb2015, PMID: 20081841 PMC4580288

[78] Uezumi A Ito T Morikawa D Shimizu N Yoneda T Segawa M . Fibrosis and adipogenesis originate from a common mesenchymal progenitor in skeletal muscle. J Cell Sci. (2011) 124:3654–64. doi: 10.1242/jcs.086629, PMID: 22045730

[79] Schmidt BA Horsley V . Intradermal adipocytes mediate fibroblast recruitment during skin wound healing. Development. (2013) 140:1517–27. doi: 10.1242/dev.087593, PMID: 23482487 PMC3596993

[80] Bochaton-Piallat ML Gabbiani G Hinz B . The myofibroblast in wound healing and fibrosis: answered and unanswered questions. F1000Res. (2016) 5:F1000 Faculty Rev-752. doi: 10.12688/f1000research.8190.1, PMID: 27158462 PMC4847562

[81] Wang K Wen D Xu X Zhao R Jiang F Yuan S . Extracellular matrix stiffness-the central cue for skin fibrosis. Front Mol Biosci. (2023) 10:1132353. doi: 10.3389/fmolb.2023.1132353, PMID: 36968277 PMC10031116

[82] Yamashita T Lakota K Taniguchi T Yoshizaki A Sato S Hong W . An orally-active adiponectin receptor agonist mitigates cutaneous fibrosis, inflammation and microvascular pathology in a murine model of systemic sclerosis. Sci Rep. (2018) 8:11843. doi: 10.1038/s41598-018-29901-w, PMID: 30087356 PMC6081386

[83] Plikus MV Guerrero-Juarez CF Ito M Li YR Dedhia PH Zheng Y . Regeneration of fat cells from myofibroblasts during wound healing. Science. (2017) 355:748–52. doi: 10.1126/science.aai8792, PMID: 28059714 PMC5464786

[84] Yoo SY Kwon SM . Angiogenesis and its therapeutic opportunities. Mediators Inflammation. (2013) 2013:127170. doi: 10.1155/2013/127170, PMID: 23983401 PMC3745966

[85] Zhao J Cao Y DiPietro LA Liang J . Dynamic cellular finite-element method for modelling large-scale cell migration and proliferation under the control of mechanical and biochemical cues: A study of re-epithelialization. J R Soc Interface. (2017) 14(129):20160959. doi: 10.1098/rsif.2016.0959, PMID: 28404867 PMC5414907

[86] Nagaraja S Chen L DiPietro LA Reifman J Mitrophanov AY . Predictive approach identifies molecular targets and interventions to restore angiogenesis in wounds with delayed healing. Front Physiol. (2019) 10:636. doi: 10.3389/fphys.2019.00636, PMID: 31191342 PMC6547939

[87] Feng H Gong S Liu J Aghayants S Liu Y Wu M . Adipose-derived stem cell exosomes: mechanisms and therapeutic potentials in wound healing. biomark Res. (2025) 13:88. doi: 10.1186/s40364-025-00801-2, PMID: 40542446 PMC12181847

[88] Biniazan F Stoian A Haykal S . Adipose-derived stem cells: angiogenetic potential and utility in tissue engineering. Int J Mol Sci. (2024) 25(4):2356. doi: 10.3390/ijms25042356, PMID: 38397032 PMC10889096

[89] Stachura A Paskal W Pawlik W Mazurek MJ Jaworowski J . The use of adipose-derived stem cells (Adscs) and stromal vascular fraction (Svf) in skin scar treatment-a systematic review of clinical studies. J Clin Med. (2021) 10(16):3637. doi: 10.3390/jcm10163637, PMID: 34441935 PMC8396936

[90] Gu P Xu A . Interplay between adipose tissue and blood vessels in obesity and vascular dysfunction. Rev Endocr Metab Disord. (2013) 14:49–58. doi: 10.1007/s11154-012-9230-8, PMID: 23283583

[91] Pastar I Stojadinovic O Yin NC Ramirez H Nusbaum AG Sawaya A . Epithelialization in wound healing: A comprehensive review. Adv Wound Care (New Rochelle). (2014) 3:445–64. doi: 10.1089/wound.2013.0473, PMID: 25032064 PMC4086220

[92] Tadokoro S Ide S Tokuyama R Umeki H Tatehara S Kataoka S . Leptin promotes wound healing in the skin. PloS One. (2015) 10:e0121242. doi: 10.1371/journal.pone.0121242, PMID: 25799398 PMC4370705

[93] Tokumaru S Sayama K Shirakata Y Komatsuzawa H Ouhara K Hanakawa Y . Induction of keratinocyte migration via transactivation of the epidermal growth factor receptor by the antimicrobial peptide LL-37. J Immunol. (2005) 175:4662–8. doi: 10.4049/jimmunol.175.7.4662, PMID: 16177113

[94] Deng Z Fan T Xiao C Tian H Zheng Y Li C . Tgf-beta signaling in health, disease, and therapeutics. Signal Transduct Target Ther. (2024) 9:61. doi: 10.1038/s41392-024-01764-w, PMID: 38514615 PMC10958066

[95] Higgins CE Tang J Mian BM Higgins SP Gifford CC Conti DJ . TGF-β1-p53 cooperativity regulates a profibrotic genomic program in the kidney: molecular mechanisms and clinical implications. FASEB J. (2019) 33:10596–606. doi: 10.1096/fj.201900943R, PMID: 31284746 PMC6766640

[96] Di X Gao X Peng L Ai J Jin X Qi S . Cellular mechanotransduction in health and diseases: from molecular mechanism to therapeutic targets. Signal Transduct Target Ther. (2023) 8:282. doi: 10.1038/s41392-023-01501-9, PMID: 37518181 PMC10387486

[97] Lampi MC Reinhart-King CA . Targeting extracellular matrix stiffness to attenuate disease: From molecular mechanisms to clinical trials. Sci Transl Med. (2018) 10:eaao0475. doi: 10.1126/scitranslmed.aao0475, PMID: 29298864

[98] Huang W Zhang Z Li X Zheng Q Wu C Liu L . Cd9 promotes tbetar2-tbetar1 association driving the transition of human dermal fibroblasts to myofibroblast under hypoxia. Mol Med. (2024) 30:162. doi: 10.1186/s10020-024-00925-5, PMID: 39333849 PMC11428569

[99] Li YY Ji SF Fu XB Jiang YF Sun XY . Biomaterial-based mechanical regulation facilitates scarless wound healing with functional skin appendage regeneration. Mil Med Res. (2024) 11:13. doi: 10.1186/s40779-024-00519-6, PMID: 38369464 PMC10874556

[100] Liggins MC Li F Zhang LJ Dokoshi T Gallo RL . Retinoids enhance the expression of cathelicidin antimicrobial peptide during reactive dermal adipogenesis. J Immunol. (2019) 203:1589–97. doi: 10.4049/jimmunol.1900520, PMID: 31420464 PMC9233297

[101] Abouzaid AM El Mokadem ME Aboubakr AK Kassem MA Al Shora AK Solaiman A . Effect of autologous fat transfer in acute burn wound management: A randomized controlled study. Burns. (2022) 48:1368–85. doi: 10.1016/j.burns.2021.10.011, PMID: 34906386

[102] Xiao Y Nie M Xu W Zhang J Lei S Wu D . The efficiency of human fat products in wound healing: A systematic review and meta-analysis. Int Wound J. (2024) 21:e70016. doi: 10.1111/iwj.70016, PMID: 39216014 PMC11365526

[103] Kanad G Ronak AP Summer EH . Cell-supplemented autologous fat grafting: A review from bench to bedside. Plast Aesthetic Res. (2024) 11:50. doi: 10.20517/2347-9264.2024.70, PMID: 39698022

[104] Grunherz L Kollarik S Sanchez-Macedo N McLuckie M Lindenblatt N . Lipidomic analysis of microfat and nanofat reveals different lipid mediator compositions. Plast Reconstr Surg. (2024) 154:895e–905e. doi: 10.1097/PRS.0000000000011335, PMID: 39480647 PMC11512614

[105] Shauly O Gould DJ Ghavami A . Fat grafting: basic science, techniques, and patient management. Plast Reconstr Surg Glob Open. (2022) 10:e3987. doi: 10.1097/GOX.0000000000003987, PMID: 35317456 PMC8932485

[106] Yu W Wang Z Dai Y Zhao S Chen H Wang S . Autologous fat grafting for postoperative breast reconstruction: A systemic review. Regener Ther. (2024) 26:1010–7. doi: 10.1016/j.reth.2024.10.007, PMID: 39553540 PMC11564784

[107] Khouri RK Khouri R-ER Lujan-Hernandez JR Khouri KR Lancerotto L Orgill DP . Diffusion and perfusion: a review of the current literature. Plast Reconstructive Surg Global Open. (2014) 2(9):e220. doi: 10.1097/gox.0000000000000183, PMID: 25426403 PMC4229279

[108] Wufuer M Choi TH Najmiddinov B Kim J Choi J Kim T . Improving facial fat graft survival using stromal vascular fraction-enriched lipotransfer: A multicenter randomized controlled study. Plast Reconstr Surg. (2024) 153:690e–700e. doi: 10.1097/PRS.0000000000010625, PMID: 37141448

[109] Zhang ZX Qiu LH Shi N Xiong SH Ma XJ Yi CG . Platelet-rich fibrin in fat grafts for facial lipofilling: A randomized, controlled split-face clinical trial. Front Surg. (2022) 9:793439. doi: 10.3389/fsurg.2022.793439, PMID: 35495758 PMC9043459

[110] Oskarsdotter K Nordgard CT Apelgren P Saljo K Solbu AA Eliasson E . Injectable in situ crosslinking hydrogel for autologous fat grafting. Gels. (2023) 9(10):813. doi: 10.3390/gels9100813, PMID: 37888386 PMC10606883

[111] He J Shi J Yang C Peng G Ju C Zhao Y . Pig-to-human lung xenotransplantation into a brain-dead recipient. Nat Med. (2025) 31(10):3. doi: 10.1038/s41591-025-03861-x, PMID: 40855190

[112] Tao KS Yang ZX Zhang X Zhang HT Yue SQ Yang YL . Gene-modified pig-to-human liver xenotransplantation. Nature. (2025) 641:1029–36. doi: 10.1038/s41586-025-08799-1, PMID: 40140580 PMC12095057

[113] Yoon S Lee S Park C Choi H Yoo M Lee SC . An efficacious transgenic strategy for triple knockout of xeno-reactive antigen genes ggta1, cmah, and B4galnt2 from jeju native pigs. Vaccines (Basel). (2022) 10(9):1503. doi: 10.3390/vaccines10091503, PMID: 36146581 PMC9505423

[114] Abbasi K Tavakolizadeh S Hadi A Hosseini M Soufdoost RS Heboyan A . The wound healing effect of collagen/adipose-derived stem cells (Adscs) hydrogel: *in vivo* study. Vet Med Sci. (2023) 9:282–9. doi: 10.1002/vms3.1059, PMID: 36571812 PMC9856998

[115] Chen X Yang R Wang J Ruan S Lin Z Xin Q . Porcine acellular dermal matrix accelerates wound healing through mir-124-3p.1 and mir-139-5p. Cytotherapy. (2020) 22:494–502. doi: 10.1016/j.jcyt.2020.04.042, PMID: 32571650

[116] Tang KC Yang KC Lin CW Chen YK Lu TY Chen HY . Human adipose-derived stem cell secreted extracellular matrix incorporated into electrospun poly(Lactic-co-glycolic acid) nanofibrous dressing for enhancing wound healing. Polymers (Basel). (2019) 11(10):1609. doi: 10.3390/polym11101609, PMID: 31623334 PMC6835469

[117] Lee D Lee J Yoon JK Kim NY Kim GW Park C . Rapid determination of perv copy number from porcine genomic DNA by real-time polymerase chain reaction. Anim Biotechnol. (2011) 22:175–80. doi: 10.1080/10495398.2011.595294, PMID: 22132811

[118] Foster DS Januszyk M Yost KE Chinta MS Gulati GS Nguyen AT . Integrated spatial multiomics reveals fibroblast fate during tissue repair. Proc Natl Acad Sci U.S.A. (2021) 118(41):e2110025118. doi: 10.1073/pnas.2110025118, PMID: 34620713 PMC8521719

[119] Hu B Sajid M Lv R Liu L Sun C . A review of spatial profiling technologies for characterizing the tumor microenvironment in immuno-oncology. Front Immunol. (2022) 13:996721. doi: 10.3389/fimmu.2022.996721, PMID: 36389765 PMC9659855

